# 1-s Productions: A Validation of an Efficient Measure of Clock Variability

**DOI:** 10.3389/fnhum.2018.00519

**Published:** 2018-12-21

**Authors:** Sarah C. Maaß, Hedderik van Rijn

**Affiliations:** ^1^Department of Experimental Psychology, University of Groningen, Groningen, Netherlands; ^2^Behavioral and Cognitive Neurosciences, University of Groningen, Groningen, Netherlands

**Keywords:** interval timing, precision, clock variance, individual differences, clinical populations

## Abstract

**Objective**: Clock variance is an important statistic in many clinical and developmental studies. Existing methods require a large number of trials for accurate clock variability assessment, which is problematic in studies using clinical or either young or aged participants. Furthermore, these existing methods often implicitly convolute clock and memory processes, making it difficult to disentangle whether the clock or memory system are driving the observed deviations. Here we assessed whether 20 repeated productions of a well-engrained interval (1 s), a task that does not incorporate memory updating nor the processing of feedback, could provide an accurate assessment of clock variability.

**Method**: Sixty-eight undergraduate students completed two tasks: a 1-s production task in which they were asked to produce a 1-s duration by ending a tone by a keypress, and a multi-duration reproduction task. Durations presented in the reproduction task were tones lasting 1.17, 1.4 and 1.68 s. No feedback was presented in either task, and the order of presentation was counterbalanced between participants.

**Results**: The observed central tendency in the reproduction task was better explained by models including the measures of clock variability derived from the 1-s production task than by models without it. Three clock variability measures were calculated for each participant [standard deviation, root mean squared residuals (RMSRs) from an estimated linear slope, and RMSR scaled by mean production duration]. The model including the scaled RMSR was preferred over the alternative models, and no notable effects of the order of task presentation were observed. These results suggest that: (1) measures of variability should account for drift; (2) the presentation of another timing task before a 1-s production task did not influence the assessment of the clock variability; and (3) the observed variability adheres to the scalar property and predicts temporal performance, and is thus a usable index of clock variability.

**Conclusion**: This study shows that just 20 repeated productions of 1 s provide a reliable index of clock variability. As administering this task is fast and easy, it could prove to be useful in a large variety of developmental and clinical populations.

## Introduction

Estimating and reproducing short intervals in the hundreds of milliseconds to seconds range is central to a wide range of behaviors. Irrespective of the theoretical framework, this type of timing is assumed to be driven by an internal time source, or clock, and memory traces of previously experienced intervals. Because of this dyad, variations in interval timing proficiency can either be driven by changes in the accuracy of the clock, or by variations in the efficacy of the memory mechanisms. Interestingly, deviations in timing performance observed in clinical populations are often attributed to variations in clock precision and accuracy (for a review see Allman and Meck, 2011). For example, Alzheimer diseased patients demonstrate increased timing variability in a bisection task using sub-second intervals (Caselli et al., 2009), and similar observations are associated with the performance of patients with bipolar disorder (Bolbecker et al., 2014) and Parkinson’s disease (Pastor et al., 1992; Harrington et al., 1998; Malapani et al., 1998). Furthermore, performance of autistic children is poorer in temporal discrimination tasks when compared with healthy, age-matched controls (Karaminis et al., 2016). Changes in temporal accuracy are, however, not limited to clinical conditions. Even during normal, healthy aging, temporal precision declines, observable by more variable timing estimations and a general decrease in accuracy (for a review see Paraskevoudi et al., 2018).

Even though these phenomena are often explained in terms of deviations in clock variance, the impairment of temporal precision may also be clock-unspecific. For example, individuals with schizophrenia display greater variability in a rhythmic tapping task, potentially caused by larger timing variability (Carroll et al., 2009). Yet, these effects have also been attributed to procedural learning (Da Silva et al., 2012), or the inability to synchronize to external events (Wilquin et al., 2018). Thoenes and Oberfeld (2017) propose general cognitive deficiencies as a potential explanation for the impaired temporal performance in individuals with schizophrenia.

Distinguishing between clock-based and more general deficiencies is difficult as most tasks that are used to assess the precision of the clock implicitly rely on a convolution of clock and memory processes. Typically, the precision of interval timing is indexed by a rhythmic tapping task or by calculating a Weber fraction (e.g., Harrington et al., 1998; Karaminis et al., 2016), a measure indicating the minimal proportional chance for a changed stimulus to be discernible from the original (“just noticeable difference”). The Weber fraction is typically calculated from a psychometric function derived from a bisection or discrimination task. In both tasks, participants have to learn either one or two comparison durations or “anchors” during the scope of the experiment. Performance in both tasks thus depends on generating the correct memory representations during the experiment itself. Moreover, calculating an accurate psychometric function requires a relatively large number of trials making it unsuitable for populations with shorter attention spans or for those that are more easily fatigued. The rhythmic tapping task on the other hand is closely linked to motor performance, a process that undergoes a separate decline in aging and certain clinical conditions (Paraskevoudi et al., 2018; PD: Jankovic, 2008). Based on similar considerations, Paraskevoudi et al. (2018) proposed that “more appropriate methods for detecting the accuracy and imprecision signatures of a slower clock are verbal estimation tasks, production tasks, and unpaced finger tapping tasks, which presumably reflect the internal tempo in its pure form” (page 11). Here, we present a first validation of a pure clock variability measure that does not incorporate memory updating during the experiment, nor the processing of feedback, and can be quickly administered.

In our study, participants were asked to produce 20 1-s intervals by ending a machine-started tone by a key-press, without feedback or prior presentation of the defined interval. As 1-s intervals are likely to be highly familiar or trained to the participants, we assumed a stable internal representation and thus attributed the variability observed in the repeated production of this interval to clock variability. The most straightforward way of determining the precision of the repeated 1-s duration is by calculating a standard deviation. This way of determining precision assumes that the accuracy of the 1-s estimation remains the same over the 20 repeated productions. However, human performance on simple tasks such as continuation tapping (e.g., Lemoine et al., 2006) is known to be subject to drifts, especially over the shorter sequences used in this study (Wagenmakers et al., 2004). These drifts are defined as slow changes of the running mean, for example, participants might speed up or slow down during the course of the experiment. Even though there is disagreement in literature about the patterns best describing long-term dependencies, it is clear that an accurate measurement of precision in the 1-s production task should account for potential drifts.

Noise is ubiquitous in human information processing (Faisal et al., 2008), and earlier work has demonstrated that humans use knowledge of their sensory variability to produce estimates that are optimal in the context of the task (e.g., Körding and Wolpert, 2004; Murai and Yotsumoto, 2018): the noisier the incoming information on a particular trial, the stronger the influence of the expectation that was built up during previous trials. Thus, the temporal precision measures derived from the 1-s task should determine how much a learnt temporal context influences the performance on a specific trial. We therefore asked participants to also complete a reproduction task of three different durations. A typical phenomenon observed in these tasks is a central-tendency effect (Hollingworth, 1910, often referred to as Vierordt’s law; see Lejeune and Wearden, 2009, for a discussion). This tendency of judgments of quantities to gravitate towards their mean, irrespective of whether it is in terms of spatial distances, durations, or any other perceptual quantity, is a highly robust perceptual effect (Jazayeri and Shadlen, 2010; Petzschner and Glasauer, 2011; Wiener et al., 2016). This effect is typically explained by assuming that the internal representation of a currently observed quantity is a mixture of the actually perceived quantity and a memory representation of all previous quantities. In the context of this study, the consequence of this “central tendency” driven by previously experienced durations is that durations shorter than this central tendency are overestimated, whereas those longer than it are underestimated.

This highly robust phenomenon that demonstrates the influence of memory on perception (see for reviews on memory influences on timing Shi and Burr, 2016 or van Rijn, 2016) can be captured in terms of Bayesian Inference (Jazayeri and Shadlen, 2010; Shi et al., 2013; see also Taatgen and van Rijn, 2011), in which the perceived duration (sensory likelihood) is integrated with previously perceived intervals that are stored in the reference memory (prior). The key feature of this process for the purpose of this work is that a narrower (or less noisy) likelihood will yield a smaller regression to the mean, and, vice versa, a stronger prior will result in more central tendency, and that the likelihood is driven by the precision of the interval timing processes. For example, in highly trained professional musicians, such as percussionists, reproduced auditory durations are reproduced to perfection, indicative of an extremely peaked likelihood, and resulting in a relatively small influence of the prior (Cicchini et al., 2012). Inversely, as aged participants exhibit more uncertainty, indicative of a wide likelihood, the influence of the prior increases, causing a stronger reliance on prior memories (Turgeon et al., 2016). In other words, the individual differences observed in the 1-s task should correlate with the central tendency observed in the reproduction task. However, both tasks rely on the production or reproduction of intervals in a similar time range. It is therefore possible that sequential effects can be observed (note that these are at a more global level than the trial-by-trial sequential effects discussed in, for example, Taatgen and van Rijn, 2011; Dyjas et al., 2012; Di Luca and Rhodes, 2016). We tested for this possibility by counterbalancing the order in which the production and reproduction task were administered.

Summarizing, we set out to test whether clock variability can be assessed with a short production task, consisting of 20 repeated productions of a 1-s interval and hypothesize that a measure of variability derived from this task should predict the amount of central tendency observed in a multi-duration reproduction task.

## Materials and Methods

### Participants

Sixty-eight undergraduate students from the University of Groningen completed the experiment in exchange for course credit. All subjects gave written informed consent in accordance with the Declaration of Helsinki. The protocol was approved by the Psychology Ethical Committee of the University of Groningen. We excluded a total of five participants based on their performance in either the production or reproduction task. One participant was excluded based on failing to follow task instructions (98% of trials failing to meet the inclusion criteria for the Reproduction task discussed below; average response time during the reproduction task was 288 ms). For the production task, four participants were excluded due to large deviations in produced durations, even after excluding the first two startup trials (i.e., out of the 67 remaining participants, 63 did not produce any intervals longer than 3 s, whereas two participants produced intervals of over 3 s on five trials, one participant on 10 trials, and one on 15 trials). In total, 63 participants remained for further analyses (mean age: 21.4, range: 17–54, SD: 5.8, 43 female).

### Apparatus

A MacBook Pro 13” (2011) controlled all experimental events. Auditory stimuli were presented through headphones (Sennheiser, HD280 Pro), with volume adjusted to comfortable levels. The experiment was programmed using Psychtoolbox-3 (Brainard, 1997; Pelli, 1997; Kleiner et al., 2007) in Matlab R2014b.

### Procedure

**Figure 1:**
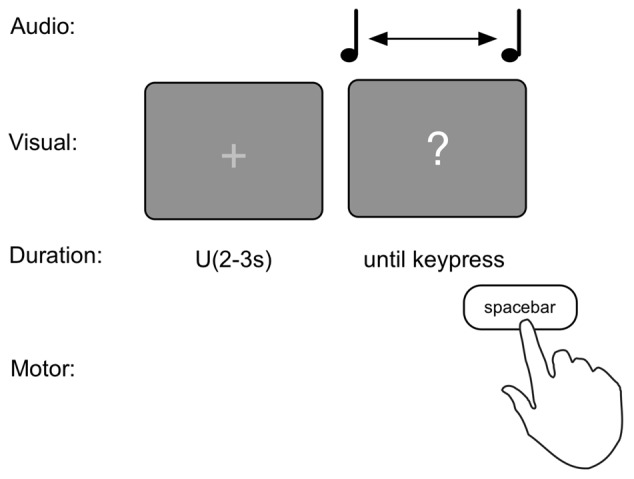
Graphical depiction of the production task.

The production task consisted of 20 trials. To prevent a rhythmic sequence, each trial commenced with an intertrial interval (ITI) with the presentation of a fixation cross “+” for a random duration between 2 s and 3 s sampled from a uniform distribution. Then a “?” appeared on the screen and simultaneously a 440 Hz pure tone started. Participants were asked to indicate when they thought 1 s since the onset of the tone had passed by pressing the spacebar (see Figure 1). As counting has been shown to increase the precision of duration judgments (Thönes and Hecht, 2017), participants were instructed to refrain from counting or keep track of time in any other way (e.g., tapping). This instruction has been shown to prevent influences of chronometric timing (Rattat and Droit-Volet, 2012).

**Figure 2:**
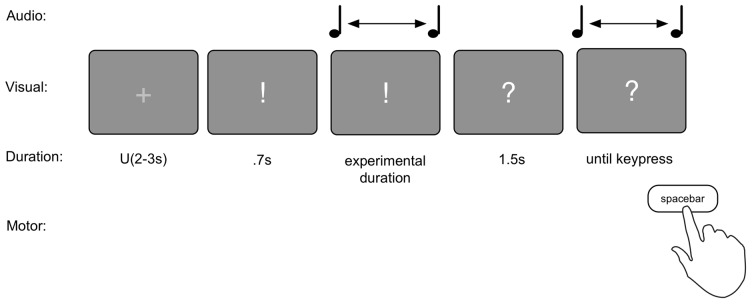
Graphical depiction of the reproduction task.

The reproduction task consisted of two blocks of 120 trials each. Each trial consisted of the presentation of a duration, and the reproduction of that duration (see Figure 2). The durations presented were 1.17, 1.4 or 1.68 s long. Each trial commenced with an ITI of a random duration between 2 s and 3 s sampled from a uniform distribution during which a fixation cross (“+”) was presented in the center of the screen. Then a “!” appeared on the screen for 700 ms to prepare the subjects for the presentation of the duration. Following this, the duration was presented by means of a 440 Hz pure tone that lasted for the duration associated with the current trial. Within each block of 120 trials, all three durations were presented 40 times, in random order. After completion of the tone, an inter stimulus interval (ISI) of 1.5 s was presented with a “?” displayed on screen. Then another 440 Hz pure tone was started. The task was to press the spacebar when the earlier presented duration had passed. To test for order effects, 31 participants performed the task in “production—reproduction” order, and 32 in reversed order (based on parity of their sequential participant number).

### Statistical Analysis

For the reproduction task, we marked trials on which response times were lower than 500 ms or greater than 2.5 s as outliers, and removed them from analyses. Of the resulting dataset, 1.4% of all data points in the reproduction task were marked as outliers.

All analyses were performed in R, with the full script and data available at the OSF^1^. The Bayesian analyses were performed with the R package BayesFactor (version 0.9.12-4.2; Morey et al., 2015) using the default prior settings, and are interpreted based on the guidelines provided by Jeffreys (1961), as adapted by Lee and Wagenmakers (2014). The reported Bayes factors summarize the extent to which an observer’s opinion of the tested variable should change based on the data. A Bayes factor of 1 indicates that both hypotheses are equally likely under the data and therefore is inconclusive. Bayes factors larger than 1 represent evidence for the alternative hypothesis of an influence of the tested independent variable on the dependent variable, and Bayes factors less than 1 represent evidence for the null hypothesis of no effect of the tested variable. For the Bayesian linear mixed effect models, we built models predicting centered estimated duration by the entered effects including participant as random factor. We then assessed the variable of interest by comparing the Bayes factor of the model including this variable with the Bayes factor associated with a model omitting this variable. To facilitate interpretation, we invert Bayes factors below 1 and describe in the text whether the Bayes factor is evidence for inclusion or exclusion of the factor. This way, all reported Bayes factors express the evidence for presence or absence of an effect as values progressively greater than 1.

## Results

### Production Task: Accuracy

**Figure 3:**
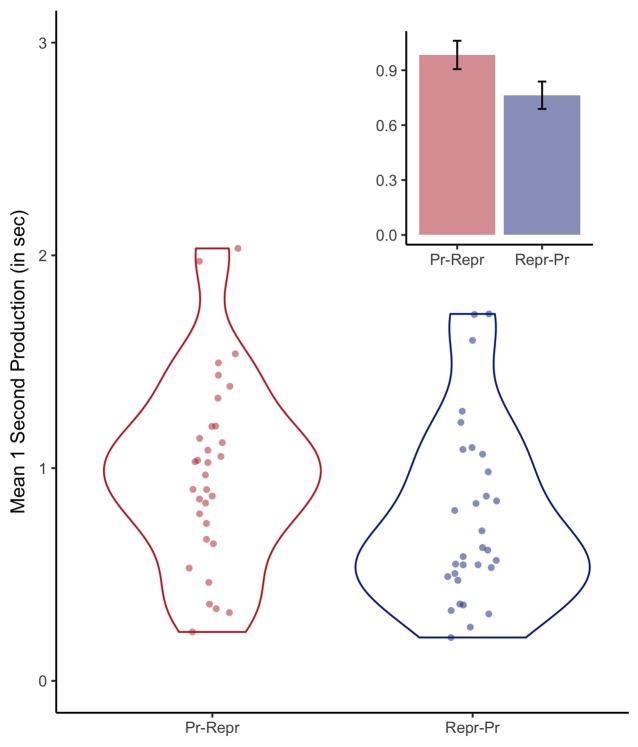
Violin plots depicting the distributions of the 1-s productions and the individual participant means, separately for the order conditions (Pr: Production, Repr: Reproduction). The inset depicts the average duration of the 1-s productions, including error bars representing standard errors of the mean with the within-participants Cousineau-Morey correction applied (Morey, 2008).

As no feedback was given during the production task, the average reproduced durations provide an index of the accuracy of the internal representation of 1-s veridical time. The first two trials were excluded, as discussed in the next section. Figure 3 depicts the average produced durations per participant and the resulting distributions as violin plots. The bar graphs depict the mean produced durations for both order conditions (0.98 s, SE = 0.08, when the production task preceded the reproduction task, and 0.76 s, SE = 0.07, for the inverse order). A Bayesian linear effect model indicated that there was no conclusive evidence either in favor or against an effect of order (BF = 1.46 ± 0.01%). However, the accuracy of the internal representation of a 1-s interval is of secondary relevance in the context of this study, as the purpose of the 1-s production task was to assess clock variability instead of (veridical) accuracy.

### Production Task: Precision

**Figure 4:**
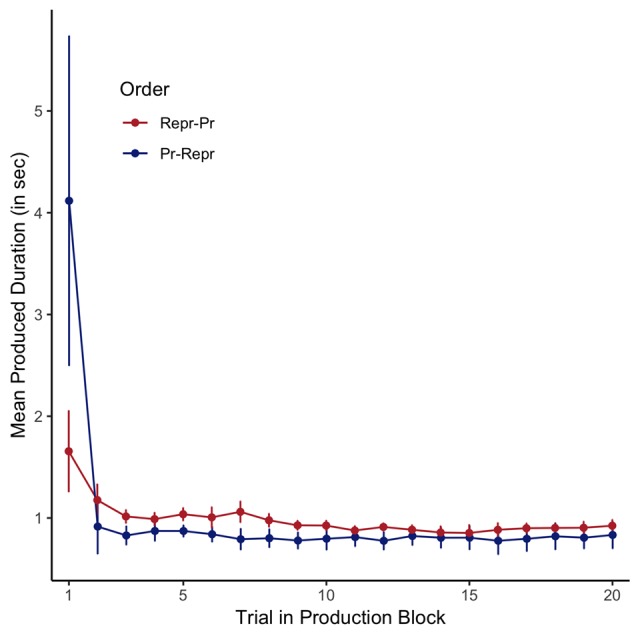
Average estimated durations in the 1-s production task, plotted per trial, separately for the order conditions (Pr: Production, Repr: Reproduction). Error bars represent standard errors of the mean with the within-participants Cousineau-Morey correction applied.

Assuming that the internal representation of a 1-s interval is firmly encoded in long-term memory, and thus not likely to be affected by a small number of production trials without feedback, the variance observed in the 1-s productions reflects the trial-by-trial clock variability. The mean trial-by-trial estimates are depicted in Figure 4, plotted separately for the two order conditions. As can be seen in this figure, the first trial is associated with very long average responses, and both the first and second trial have noticeably bigger error bars than the following trials. In all subsequent analyses (and the analyses reported in the previous section), we have therefore considered these two initial trials to be “start-up trials,” and excluded them from further analysis.

**Figure 5:**
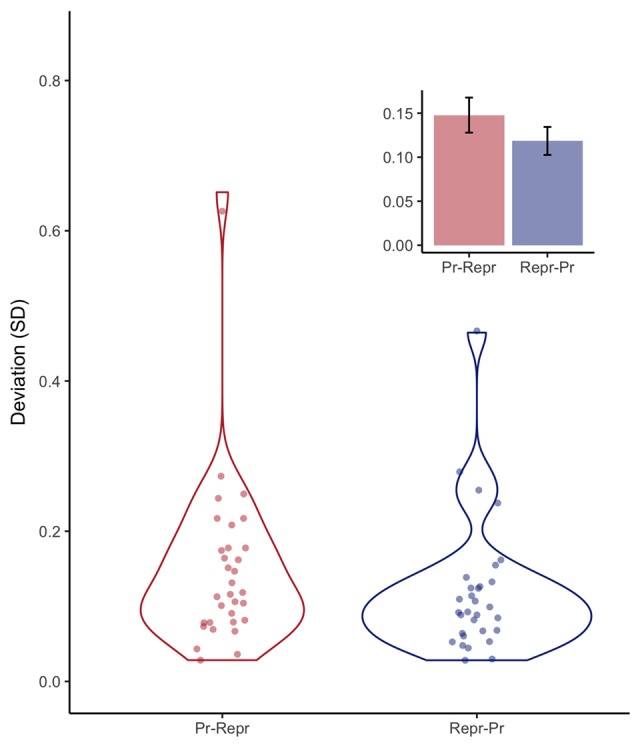
Violin plots depicting observed standard deviation per participant, and the resulting continuous distributions, separately for the order conditions (Pr: Production, Repr: Reproduction). Inset depicts mean deviation with error bars representing standard errors of the mean with the within-participants Cousineau-Morey correction applied.

The most straightforward measure of this variability is the variance or standard deviation. Figure 5 shows, again for both order conditions, the standard deviation of the final 18 trials of the sequence of 20 trials. A Bayesian linear model provided anecdotal evidence against order having an influence on the deviation expressed in SD (BF = 2.23 ± 0%).

**Figure 6:**
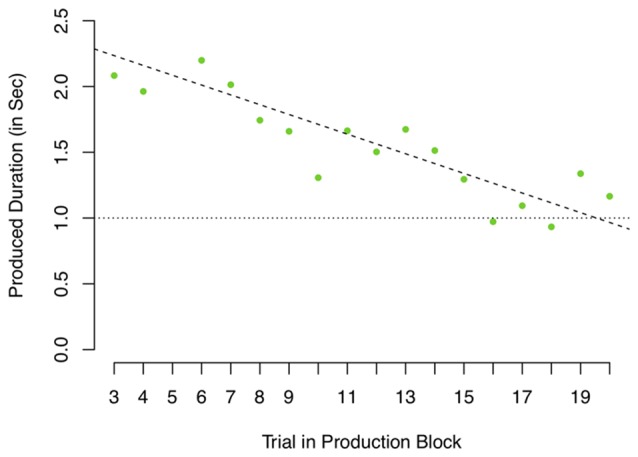
Produced durations in the 1-s production task of one participant. Dashed line depicts linear regression predicting produced duration by trial.

The standard deviation is an appropriate measure if the noise can be assumed to be centered around a fixed mean. However, if the repeated samples are drawn from a distribution of which the mean changes over time, the standard deviation calculated assuming a fixed mean will overestimate the true variance as the shift in mean will increase the standard deviation. In the context of this task, if the internal representation of a 1-s interval shifts, the standard deviation will overestimate the clock’s noisiness. Figure 6 depicts the 1-s production data of a single participant. As can be seen, this participant shows a slow drift from productions of around 2 s to productions of 1 s, but actually shows relatively little variance around this estimated trend. Assuming slow drifts in performance, calculating a standard deviance would overestimate this participant’s clock noise. Even though Figure 4 might suggest an overall slope close to 0 (the mean of individual slopes is −0.0055), the range is relatively large (−0.08 to 0.04, reflecting a drift of −1,440 to 720 ms between trial 3 and 20). As there is decisive evidence for a drift (one sample Bayesian *t*-test on the absolute slopes as depicted in Figure 6, BF = 4.83 × 10^4^ ± 0%), a reliable measure should account for drift when estimating clock variance. We therefore also calculated the root mean squared residuals (RMSRs) based on a linear regression predicting produced duration as a function of trial number (coded as 1–18, after removing the first two trials) fitted separately for each participant.

**Figure 7:**
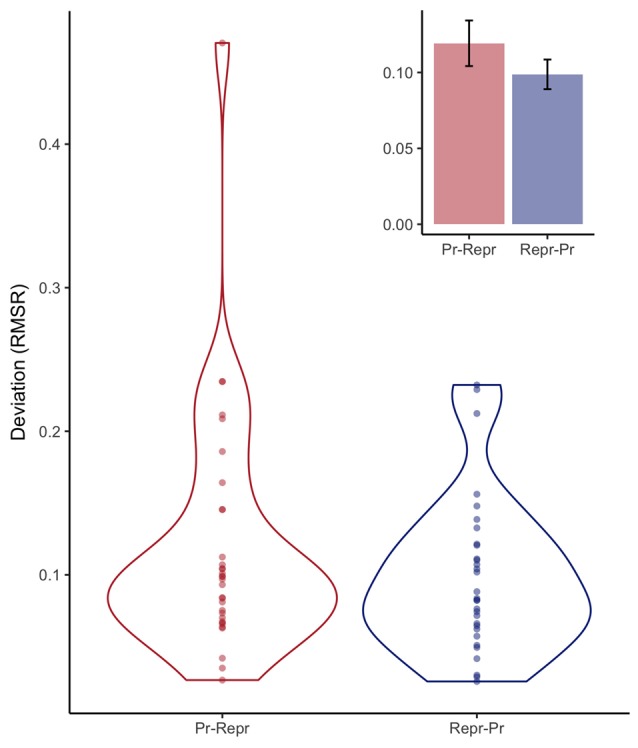
Violin plots depicting observed deviation expressed in root mean squared residual (RMSR) from a linear fit estimated per participant, and the resulting continuous distribution, separately for the order conditions (Pr: Production, Repr: Reproduction). Inset depicts mean deviation with error bars representing standard errors of the mean with the within-participants Cousineau-Morey correction applied.

Figure 7 depicts the RMSR for the two order conditions, analogous to the way the SD was plotted in Figure 5. As with the SD measures, a Bayesian linear model provided anecdotal evidence against order having an influence on the deviation expressed in RMSR (BF = 2.26 ± 0%).

### Clock Variability

**Figure 8:**
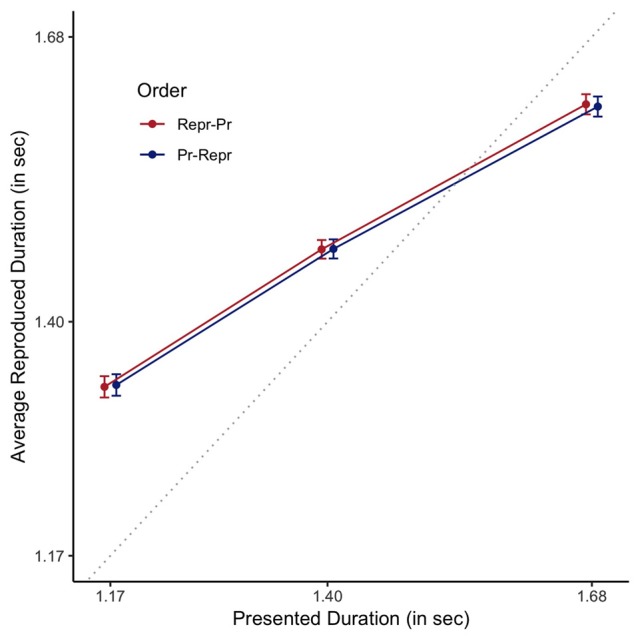
Reproduced durations as a function of presented duration, plotted staggered on the horizontal axis, separately for the order conditions (Pr: Production, Repr: Reproduction). Error bars are standard errors of the mean with the within-participants Cousineau-Morey correction applied. The dotted line represents veridical time.

To assess which of the proposed measures is a better estimate of the clock variance, we assessed whether a participant’s standard deviation or RMSR was a better predictor of the central tendency observed in a reproduction task. The data of the multi-duration reproduction task is graphically depicted in Figure 8.

**Figure 9:**
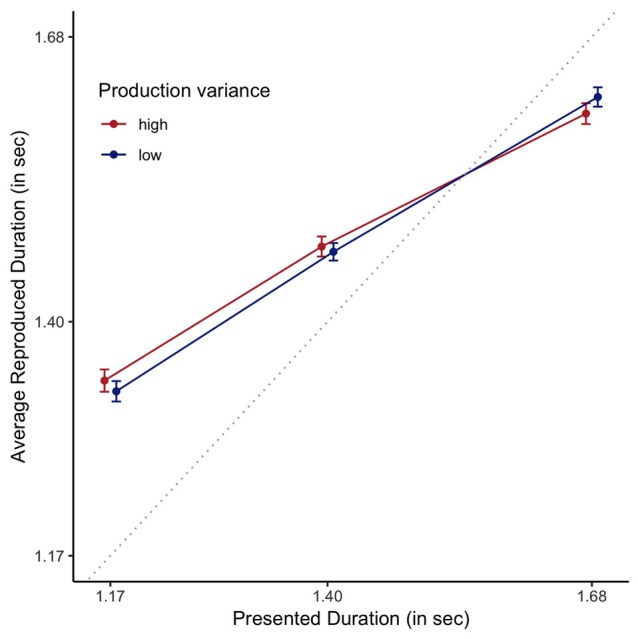
Reproduced durations as a function of presented duration, plotted staggered on the horizontal axis and plotted based on a median split on RMSR variance. Error bars are standard errors of the mean with the within-participants Cousineau-Morey correction applied. The dotted line represents veridical time.

For the analyses of this data, we centered both presented and reproduced duration by subtracting 1.4 s from the presented and reproduced durations. For the presented durations, this ensures that the predictors have mean 0, making it easier to interpret the effects of additional predictors. Moreover, and this holds for both presented and reproduced durations, it allows for easier interpretation of resulting coefficients. As a baseline model, we fitted a Bayesian linear mixed effect model, with participant as random factor, predicting centered estimated duration by centered presented duration. Evidence in favor of the more complex model that included presented duration was decisive when compared to a model just including an intercept (BF = 9.17 × 10^1244^ ± 7.99%). Comparing this model to a model that also included experimental order provided decisive evidence against the more complex model (BF = 407.24 ± 14.59%). Based on the rationale that clock variability should influence the width of the likelihood, and as such the relative contribution of the prior, the best estimate of the veridical clock variability should best predict reproduced duration. Decisive evidence was obtained for the inclusion of each of the clock variance measures when compared to the simpler model that did not include any estimate of clock variance (BF = 4.48 × 10^10^ ± 9.74% for the SD-based measure, and BF = 2.47 × 10^13^ ± 9.75%, for the RMSR-based measure), demonstrating that clock variability as estimated by a 1-s production task does indeed predict the amount of central tendency in a multi-duration reproduction task. This is depicted in Figure 9, where participants with higher clock variance also demonstrate a stronger central tendency effect. More importantly, a Bayesian model including RMSR is a decisively better predictor of the estimated durations than a model including the SD-based measure (BF = 5514.79 ± 7.9), demonstrating the superiority of the new RMSR measure.

One of the central findings in interval timing is that temporal precision is relative to the duration of the interval being estimated, a phenomenon called the scalar property of variance. As Figure 3 demonstrates, the average produced durations range from a couple of hundred milliseconds to approximately 2 s. Scaling the observed variances by the mean produced duration would result in a less biased estimate of the internal noisiness of the clock. To test this assumption, we fitted another Bayesian model that included the RMSR variance divided by the mean produced duration. This scaled model is a decisively better predictor of the estimated durations than a model including the non-scaled RMSR measure (BF = 4.15 × 10^7^ ± 13.39%).

**Figure 10:**
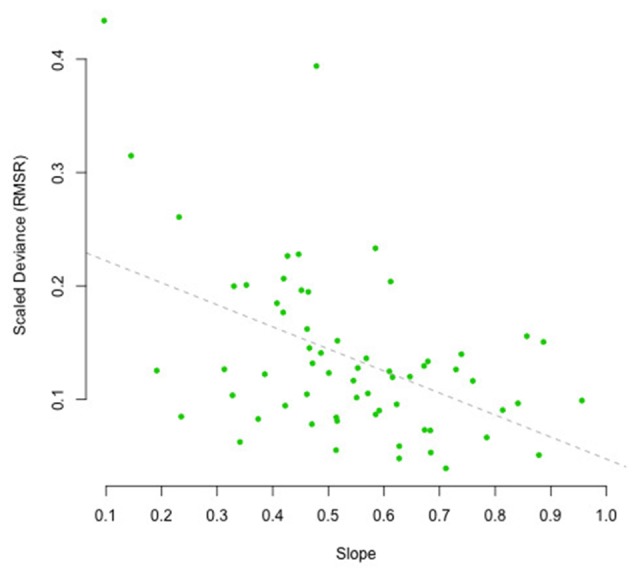
Scaled deviation (RSMR) as a function of the estimated slope of the regression towards the mean as observed in the reproduction task, plotted per participant. The dashed line depicts the regression line.

To assess the influence of the scaled RMSR measure on the regression towards the mean, we have plotted the estimated slope of the regression line as shown in, for example, Figure 8, against the scaled RMSR (see Figure 10). A slope of 1 would indicate a participant, whose reproductions are not at all influenced by context, whereas a slope of 0 would indicate a participant who always reproduces the exact same duration, irrespective of the duration of the presented interval. Or, in Bayesian inference terms, a slope of 1 indicates complete reliance on the likelihood, whereas a slope of 0 indicates complete reliance on the prior. As expected, increased clock variance is associated with smaller slopes, and vice versa: results of the Bayesian correlation indicate extreme evidence (BF = 325.58 ± 0%) in favor of a large or moderate negative association between the scaled RSMR measure and the slope, expressing the regression towards the mean [*r* = −0.45, MAD = 0.11, 90% CI (−0.63, −0.25)].

## Discussion

The goal of this article was to assess whether clock variability could be reliably measured in a span of a couple of minutes so that it can be applied in both prototypical experimental populations (i.e., young adults) and clinical or developmental studies in which complex or lengthy experiments are often problematic. We determined clock variability in a simple production task, and demonstrate that this variability predicts the central tendency observed in a multi-duration reproduction task. Following the rationale of Bayesian inference, this indicates that the measured variance in the production task is related to the width of the likelihood distribution in the multi-duration reproduction task, which has been associated with the noise in the clock parts of the temporal system (e.g., Shi et al., 2013). Thus, we can assume that the assessed variance in the production task, which takes less than 3 min to administer, is a reliable index of clock variance. Here, we will first discuss a number of methodological issues related to this paradigm and experiment, and then discuss a number of more theoretical considerations.

We asked the participants to produce 1-s durations by ending a tone 1 s after onset by a keypress. We explicitly opted for this duration as we assumed that typical participants will have a reasonably stable and well encoded representation of a second duration, due to the prevalence of this duration in everyday life. Because of this well ingrained duration, participants will hopefully be able to produce this duration without too much effort, and at the same time, it is unlikely that 20 repeated productions of this duration, without any external feedback, would cause noticeable changes in the internal representation.

Before conducting this experiment, we did not not have specific information on whether it would be necessary to exclude a number of initial trials as “start-up trials,” but as the first two trials were clearly associated with longer and more variable (between participants) produced durations, we categorized these first two 1-s productions as start-up trials. However, the population tested in this experiment are young adults who were well trained in participating in psychophysiological experiments, making it likely that more start-up trials might be needed when this paradigm is administered in other populations. Also, we considered the possibility that order effects might influence performance in either the production, or reproduction task. For example, after performing two blocks of the reproduction task, which contained durations between 1 s and 2 s, the 1-s estimates could be affected. However, Figure 3 does not show any hints of the reproduction task influencing the 1-s production task (the numerical effects are in opposite direction as what we be expected), and the Bayesian analyses did not provide any reliable evidence for a difference between the two order conditions. This indicates that the 1-s estimations are immune to perturbations from the multi-duration reproduction task used in this study.

To quantify the clock variability, we assessed the predictive power of different measures of variance. As the produced durations show clear drifts during the 20 trials even though no feedback was provided, standard measures of variance that assume a fixed mean would overestimate clock variability. We therefore fitted a linear model to the produced duration of each individual, and calculated a deviation measure by taking the RMSRs of this linear model. Obviously, it is not unlikely that the drift in produced durations follows a more complex pattern than is captured by a simple linear regression (see, for example, the discussions on short range dependencies in Wagenmakers et al., 2004). However, fitting more complicated patterns would quickly result in overfitting given the limited number of trials acquired in this task. We therefore refrained from estimating more complex patterns. When the different variability measures were contrasted, the linear-model-based RMSR measure provided the best prediction of the central tendency observed in a multi-duration production task. As variance is known to be linearly related to the length of the durations produced (scalar property see e.g., Gibbon et al., 1984), we also tested the predictive power of a model in which we divided the RMSR by the produced durations. This scaled RMSR measure outperformed the non-scaled RMSR variance measure, indicating that the measured variance adhered to the scalar property. Bayesian models of time estimations (e.g., Jazayeri and Shadlen, 2010) have demonstrated that the perception of longer durations are reflected in wider likelihoods than those associated with shorter intervals. Here, we demonstrate that a similar effect can be observed between participants: the higher the variance of participants’ performance during the 20 repeated 1-s productions, the more their reproduced durations will be affected by the prior.

One potential caveat of this method is that the observed variance in the 1-s production task may be driven by motor noise, which would also result in a stronger reliance on the prior in the reproduction task. This potential convolution of motor and clock noise is a challenge in studies assessing clock variability (see, for example, Cicchini et al., 2012; Turgeon et al., 2016, for discussion). If motor noise would be the driving factor, noise should be independent of produced durations, as all durations have the same motor action (i.e., the keypress indicating the end of the interval). Conversely, if the observed variability is driven by a noisy clock, the amount of variability should be directly related to the length of the produced duration. As the variance measure that accounts for produced length, the scaled RMSR, resulted in the best fit, we do not find support for the notion that motor noise is driving the observed phenomena. Yet, a future study could independently measure a participant’s motor noise to separately assess its contribution to the central tendency effect.

In this manuscript, we assess whether we can measure clock variance in a short, 20-trial production task. This observed statistic predicts central tendency in a multi-duration task, a phenomenon known to be dependent on clock variability. Another way to assess the reliability of the measured clock variability is to compare the scaled RMSR measure with the Weber fraction, a measure typically used when clock variability is estimated. However, as Weber fractions are derived from paradigms that rely on memory representations learned during the experimental session, the Weber fraction is indicative of the noisiness of the whole temporal system (but note that experimental or pharmacological manipulations might allow separating both influences, e.g., Meck, 1983). Therefore, it would be useful to determine Weber fractions based on paradigms that vary in their reliance on memory processes to determine the consistency of estimated clock variance statistics. At the same time, additional research is needed to relate the here presented 1-s production method to other established measured of clock variability.

To conclude, we have shown that just 20 trials of a 1-s production task result in a reliable measure of clock variance. The observed variability adheres to the scalar property and predicts temporal performance observed in a reproduction task. As no feedback is required, and memory processes are unlikely to play an important role in this paradigm, this clock variance measure can be used to disentangle the extent to which temporal behavior in a task is driven by memory processes or clock deviations. With its fast and easy application, this task is suitable to be implemented in clinical and various developmental populations, even in attention-span limited participants. Hopefully, this task can be a useful addition to the toolkit of researchers interested in unraveling the locus of deviations found in temporal performance.

## Data Availability

The datasets analyzed for this study can be found in the Open Science Framework (https://osf.io/bhe97/?view_only=9c3ea605d482416691b43588fecf92f5).

## Author Contributions

SM and HR conceived the experiment, analyzed the data, and wrote the manuscript. SM collected all data.

## Conflict of Interest Statement

The authors declare that the research was conducted in the absence of any commercial or financial relationships that could be construed as a potential conflict of interest.

## Footnotes

^1^https://osf.io/bhe97/?view_only=9c3ea605d482416691b43588fecf92f5
